# Awareness and use of tobacco products among underage individuals: findings from the altria client services underage tobacco use survey 2020–2022

**DOI:** 10.1186/s12889-023-15610-1

**Published:** 2023-04-07

**Authors:** Hui G. Cheng, Andrea R. Vansickel, Edward G. Largo

**Affiliations:** Altria Client Services LLC, 601 E. Jackson, Richmond, VA 23219 USA

**Keywords:** Underage Tobacco Use, Survey, United States

## Abstract

**Background:**

Tobacco use among underage individuals is a public health concern. Timely data about tobacco products, especially emerging products such as novel oral nicotine products (NPs), can provide critical information for the prevention of underage tobacco use. With a recent federal law raising the legal age of purchase of tobacco products from 18 to 21, it is of interest to benchmark awareness and use of tobacco products in the new underage population, young adults 18–20 years old. This study provides estimates on awareness and use of tobacco products among underage individuals 13–20 years old during May 2020 to August 2022 in the United States.

**Methods:**

Altria Client Services Underage Tobacco Use Survey (UTUS) is a repeated cross-sectional survey conducted every quarter-year. A stratified random sampling approach was used to draw nationally representative samples of household dwelling individuals 13–20 years old. Information about the awareness and use of tobacco products was obtained via online self-administration or phone interviews after a consent/assent process.

**Results:**

A sizable portion of underage individuals were aware of NPs (~ 40% among youth and ~ 50% among underage young adults), although past 30-day use was low (< 2%). The lowest levels of awareness and use were observed for heated tobacco products and snus. E-cigarettes were the most used tobacco products among underage individuals. Underage young adults (i.e., 18–20 year olds) were more likely to use tobacco products than youth (i.e., 13–17 year olds). There was no substantial change over time in the awareness and use of tobacco products during the study period despite a slight increase in past 30-day prevalence of e-cigarette use among youth between quarter 1 of 2021 and quarter 2 of 2022.

**Conclusions:**

The awareness and use of tobacco products remained relatively stable between May 2020 and August 2022. There is a notable level of awareness of novel NPs among underage individuals.

**Supplementary Information:**

The online version contains supplementary material available at 10.1186/s12889-023-15610-1.

## Background

The United States (US) Surgeon General declared youth vaping an epidemic in 2018 [1]. National cross-sectional studies have consistently demonstrated a rapid, increasing trend in youth e-cigarette use alongside a steadily decreasing trend in all other youth tobacco use between 2011 and 2019 [2, 3]. The rise in youth e-cigarette prevalence reached an inflection point recently. According to the National Youth Tobacco Survey, prevalence of e-cigarette use among high school students dropped from 27.5% to 2019 to 19.6% in 2020 (pre-COVID-19 pandemic); the most recent estimate was 11.3% in 2021 (during the COVID-19 pandemic) [4–6]. Timely market-relevant data will be crucial to preventing any future surges in youth tobacco use.

In the meantime, two novel tobacco product categories, oral nicotine products (NPs), which do not contain tobacco leaf, and inhalable heated tobacco products (HTPs), products that heat but do not burn tobacco, have recently emerged in the US market. IQOS®, an HTP, was the first of its kind authorized by FDA for sale in the US in April 2019. Based on data from the 2017 International Tobacco Control Youth Tobacco and E-cigarette Survey, however, 9.1% of 16–19 year olds in the US were aware of IQOS® at that time [7]. Data from the 2019 National Youth Tobacco Survey (NYTS), a US national school-based survey, revealed that 12.8% of US middle- and high-school students were aware of HTPs; 2.4% had ever used an HTP, and 1.6% had used an HTP in the past 30 days [8]; data from the 2020 NYTS showed a similar past 30-day prevalence (1.4%) [4]. As for 18–20 year olds, data from the 2019 Tobacco Use Supplement to the Current Population Survey showed a similar level of awareness of HTPs (13.3%) but lower level of ever use (0.96%) compared to estimates for youth based on NYTS data [9]. With respect to NPs, the NYTS added questions related to nicotine pouches in 2021 which showed that 1.9% of high school and middle school students ever used nicotine pouches and 0.8% reported current use (Gentzke, Wang et al. 2022).

In addition to new, emerging tobacco product categories, the federal minimum legal age to purchase tobacco in the U.S. has recently changed from 18 to 21 years (i.e., in December 2019), creating a new underage population of 18–20 year old individuals. Evidence suggests, at least at the state and local level, that raising the minimum legal age to purchase tobacco to 21 years reduces cigarette smoking and vaping among 18–20 year olds [10–12]. Current school-based national surveys (e.g., NYTS, Monitoring the Future, and the Youth Risk Behavior Surveillance System) are not well-poised to evaluate tobacco use among the 18–20 year old population as they may not provide sufficient coverage of this population.

Given the rapidly evolving underage tobacco use landscape and to complement existing government surveys, Altria Client Services (ALCS) launched the ALCS Underage Tobacco Use Survey (UTUS) in May of 2020 to enhance the ability to obtain timely information related to tobacco use that covers a broad range of traditional and novel tobacco products among underage individuals, including the new underage population of 18–20 year olds. The UTUS is a repeated cross-sectional survey that collects data on underage tobacco use every quarter year. Here we present findings from the UTUS between May of 2020 (the second quarter of 2020) and August of 2022 (quarter 3, 2022). The data collection period coincides with the COVID-19 pandemic, including a period when multiple national government surveys were disrupted (quarters 2–4, 2020). The main aim of this initial report was to provide data over time on three major tobacco-use-related milestones (i.e., awareness, ever use, and past 30-day use) of various tobacco products among underage individuals 13–20 years of age during May 2020 to August 2022.

## Methods

### Study Period, Study Population, and sampling methods

UTUS was launched in May of 2020 and has been conducted on a quarter-year schedule. Data from the second quarter of 2020 to the third quarter of 2022 were used in this analysis (see Table 1 for more details). The study population was household dwelling individuals 13–20 years of age. Samples were drawn using a list-assisted, address-based, stratified, random sampling method. The primary sample units were residential housing units in the US, including the 50 states and the District of Columbia, obtained from the US postal service computerized delivery sequence file, and flagged as likely to contain a person 13–20 years of age. First, housing units’ addresses on the sampling frame were stratified by geographic regions, urban/rural residence, and three age groups (13–15, 16–18, and 19–20 years old). Geographic regions were the four census regions (i.e., Northeast, Midwest, South, and West). The South region was further divided into four subregions – Atlanta (Georgia), Charlotte (North Carolina), Richmond (Virginia), and the rest of the South region for surveys conducted during quarter four of 2020 to quarter one of 2022 to fulfill postmarket surveillance requirements as mandated by the FDA.

Within each stratum, a random sample of addresses was drawn. Invitation letters were sent to all sampled addresses to ask an adult household member to enumerate all household members. Up to two eligible individuals were randomly selected from each household to participate in the survey. For potential participants 13–17 years of age, parental/guardian consent and participant assent were required. For potential participants 18–20 years of age, participant consent was required. The consent/assent form contained information about the purpose of the study, the survey procedure, potential risks and benefits, steps to protect privacy, the voluntary nature of participation, compensation for completing the survey, and contact information of the Institutional Review Board (IRB). In the consent/assent form, it was stated that the survey was sponsored by a tobacco manufacturer. After proper consents and assents were obtained, the selected individuals were directed to complete the questionnaire. Participants received a code to obtain a $20 e-gift card from Amazon or Target upon completion of the survey. For participants 13–17 years of age, a parent code was necessary to obtain the e-gift-card. The study was approved and overseen by Sterling IRB (Protocol #: ALCS-REG-20-10-CCR).

### Assessment

Most respondents completed the survey via online self-administration (99%) with a few completed via phone interviews (1%) in English or Spanish. The survey consisted of modules for the following nine tobacco product categories: ecigarettes, cigarettes, cigars (cigars, cigarillos, or little cigars), smokeless tobacco (chewing tobacco, snuff, or dip), hookah, pipe tobacco, snus, HTPs, and NPs (see Supplementary Table 1 in Additional File for description of each tobacco category used in the survey). Each module contained questions about the awareness and use of the tobacco product category. Proper skips were implemented to reduce respondent burden. Survey questions were largely sourced from national surveys such as the NYTS and the Population Assessment of Tobacco and Health (PATH). The questionnaire is available at https://sciences.altria.com/library/underage-tobacco-use-survey?src=topnav.

Questions about the awareness of a tobacco product were in the form of “have you ever seen or heard of … before this study?” Questions about ever use of a tobacco product were in the form of “have you ever used …, even once or twice?” or “have you ever smoked…, even one or two puffs?” A “yes” answer to respective questions was coded as being aware of, or having ever used, the tobacco product; a “no” answer was coded as not being aware of, or having never used, the tobacco product.

Questions about the recency of use were in the form of “when was the last time you used …, even one or two times?” or “when was the last time you smoked a …, even one or two puffs?” Respondents who answered “earlier today,” “not today, but sometime during the past 7 days,” or “not during the past 7 days, but sometime during the past 30 days” were coded as past 30-day users, and those who answered “not during the past 30 days but sometime during the past 6 months,” “not during the past 6 months but sometime during the past year,” “1 to 4 years ago,” “5 or more years ago,” or indicated they had never used the respective tobacco product were coded as non-past-30-day users.

### Analysis

All estimates were weighted to account for selection probabilities and non-response patterns, and post-stratification was used to bring the sample into balance with the target sample with respect to age, sex, race/ethnicity, geographic region, and urban/rural residency. To account for potential clustering within households and the stratified sampling approach, variance was computed using Taylor series approximation. Stata 16.0 (College Station, TX) was used for analysis.

## Results

Table 1 shows the schedule and final sample size of each survey. The quarter four survey of 2020 was designed to be a double-sample size survey to enhance the annual sample size. A total of 14,708 individuals 13–20 years of age completed the survey during May 2020 to August 2022 (n = 8,650 and 6,058 for 13–17 and 18–20 year olds, respectively). Response levels varied from 6.5 to 8.6% at the household screening stage (i.e., screener completion among all invited households) and 54–61% at the individual interview stage (i.e., survey completions among individuals who were sampled; see Table 1 for more details).

**Table 1:** Altria Client Services Underage Tobacco Use Survey Schedule and Sample Sizes

Year	UTUS wave	Duration	Sample size		Response level	
			13–17	18–20	Household screening*	Individual interviews*
2020	Quarter 2	May – Jun.	857	632	8.6%	61%
	Quarter 3	Jul. – Aug.	647	380	7.0%	58%
	Quarter 4	Oct. – Nov.	1,637	1,099	7.2%	60%
2021	Quarter 1	Jan. – Feb.	966	721	8.4%	60%
	Quarter 2	Apr. – May	706	505	6.5%	60%
	Quarter 3	Jul. –Aug.	692	492	6.6%	59%
	Quarter 4	Oct. – Nov.	770	535	6.7%	59%
2022	Quarter 1	Jan. – Feb.	889	621	7.7%	59%
	Quarter 2	Apr. – May	734	537	7.8%	55%
	Quarter 3	Jul. –Aug.	752	536	8.0%	53%

* Response level at household screening is defined as the number of households that completed the household screener divided by the number of invitations sent. Response level at the individual interview level is calculated by the number of individuals who completed the survey divided by the number of individuals selected to complete the survey

**Fig. 1:**
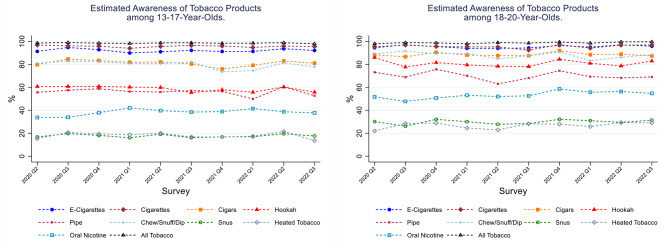
Estimated Awareness (weighted %) of Tobacco Products. Data from the ALCS UTUS, May 2020 to August 2022. Panel (1) Estimated Awareness of Tobacco Products among 13-17-year-olds. Panel (2) Estimated Awareness of Tobacco Products among 18-20-year-olds

Selected demographic characteristics of the UTUS sample are shown in Table 2. Figure 1 presents the estimated awareness of various tobacco products assessed. (Estimates and their 95% confidence intervals are in Supplementary Table S2 in Additional File.) Almost all underage individuals were aware of e-cigarettes and cigarettes (> 90%). In contrast, less than a quarter of youth (13–17 year olds) and less than a third of underage young adults (18–20 year olds) had heard of, or seen, snus or HTPs. With respect to NPs, of those surveyed, more than a third of youth and more than half of underage young adults had heard of, or seen, these products. Underage young adults were more likely to be aware of tobacco products compared to youth, except for e-cigarettes and cigarettes. There was a slight increasing trend (from 33.7 to 42.1%) in the awareness of NPs among youth until the first quarter of 2021, which plateaued afterwards.

**Table 2:** Demographic Characteristics of Study Sample. Data from the ALCS UTUS, May 2020 to August 2022

	n	Weighted %	95%CI
Gender			
Male	7,178	51.3	(50.3, 52.3)
Female	7,530	48.7	(47.7, 49.7)
Age Group			
13–17	8,650	65.9	(65.0, 66.8)
18–20	6,058	34.1	(33.2, 35.0)
Race/Ethnicity			
Hispanic	3,395	25.2	(24.2, 26.2)
Non-Hispanic white	8,386	50.5	(49.4, 51.7)
Non-Hispanic black	1,888	13.5	(12.7, 14.4)
Non-Hispanic others	1,039	10.8	(10.0, 11.7)
Census Region			
Northeast	2,002	15.7	(15.2, 16.2)
Midwest	3,319	20.9	(20.5, 21.3)
South	6,347	39.1	(38.5, 39.7)
West	3,040	24.3	(23.8, 24.8)
Urban/Rural Residency			
Urban	12,432	85.8	(85.4, 86.3)
Rural	2,276	14.2	(13.7, 14.6)

**Fig. 2:**
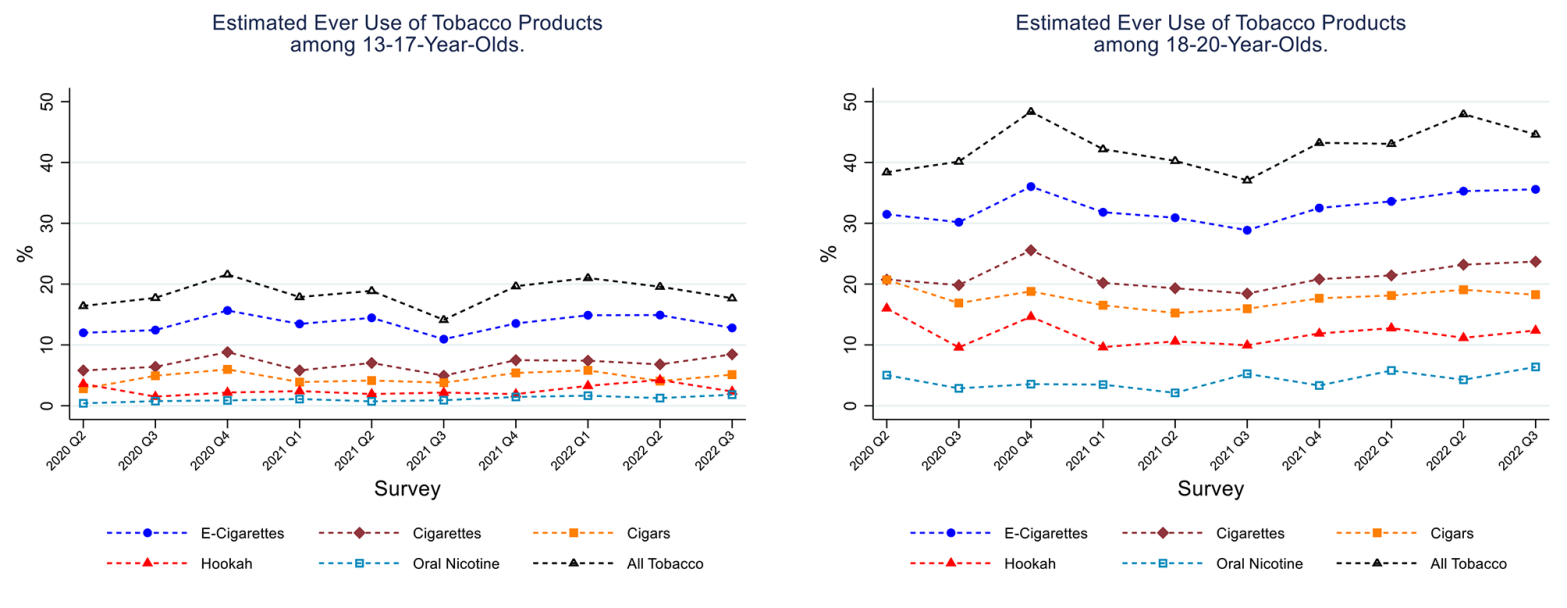
Estimated Ever Use (weighted %) of Tobacco Products. Data from the ALCS UTUS, May 2020 to August 2022. Panel (1) Estimated Ever Use of Tobacco Products among 13-17-year-olds. Panel (2) Estimated Ever Use of Tobacco Products among 18-20-year-olds

**Fig. 3:**
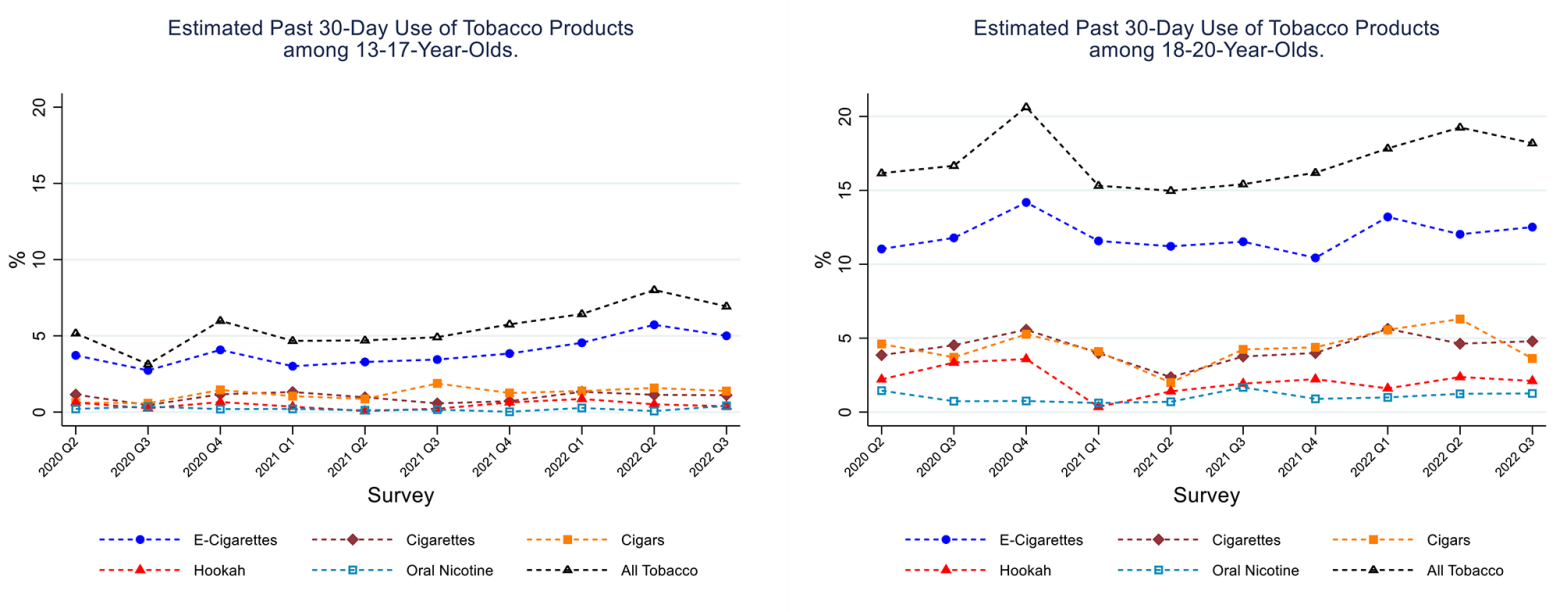
Estimated Past 30-Day Use (weighted %) of Tobacco Products. Data from the ALCS UTUS, May 2020 to August 2022. Panel (1) Estimated Past 30-Day Use of Tobacco Products among 13-17-year-olds. Panel (2) Estimated Past 30-Day Use of Tobacco Products among 18-20-year-olds

Figure 2 (selected tobacco products) and Table S3 (all tobacco products under study; Supplementary Table S3 in Additional File) present estimated ever use of tobacco products. E-cigarettes were the most commonly used tobacco products among both youth and underage young adults during the study period. Between 10% and 15% of youth had ever tried an e-cigarette, and approximately a third of underage young adults had tried an e-cigarette. Between 5 and 10% of youth and 15–25% of underage young adults had tried cigarettes or cigars. Underage young adults were more likely to have tried the tobacco products assessed, compared to youth. Similar patterns were observed for the past 30-day use of tobacco products (Fig. 3 and Table S4). E-cigarettes were the most commonly used tobacco product in both youth (3–5%) and underage young adults (10–15%). There was a slight increase in past 30-day e-cigarette use between quarter 1 of 2021 (3.0%; 95% CI = 2.6%,5.7%) and quarter 2 of 2022 (5.7%, 95% CI = 3.9%, 8.3%) among 13–17 year olds, which plateaued in the most recent quarter (i.e., 5.0%; 95% CI = 3.6%, 6.8% in quarter 3 of 2022). Approximately 5% of underage young adults smoked cigarettes or cigars in the 30 days prior to the assessment.

## Discussion

In this study, we provide data from a national survey conducted during the COVID-19 pandemic and found that (a) a sizable portion of underage individuals were aware of emerging tobacco products, such as HTPs and oral NPs; (b) there was no substantial change in the awareness and use of tobacco products during the study period (i.e., May 2020 to August 2022); (c) e-cigarettes were the most commonly used tobacco products among underage individuals; and (d) underage young adults were more likely to use tobacco, compared to youth.

To the best of our knowledge, this is among the first studies to report the awareness and use of emerging NPs, such as nicotine pouches, among household dwelling individuals 13–20 years of age. NYTS, a school-based national survey of middle- and high-school students added nicotine pouches in the assessment in 2021. The estimated awareness of nicotine pouches from this study is comparable to those from the NYTS [13], and both surveys showed low past 30-day prevalence of nicotine pouch use [14, 15]. In addition, the current study provided estimates for underage adults 18–20 years of age. In this study, we found that despite an intermediate level of awareness (as compared to other tobacco products), use of such products remained at a low level during the study period (i.e., past 30-day use < 0.5% and < 1.5% among youth and underage young adults, respectively). Although still a small share of overall tobacco consumption, nicotine pouches have become one of the fastest growing tobacco product categories in the US [16]. Our findings suggest no substantial uptake of these new NPs; however, the moderate levels of awareness suggest that continued active monitoring is necessary.

HTPs are another emerging tobacco product category. A few studies have reported the awareness and use of HTPs [4, 8, 9, 14, 15]. The current study provides more recent and finer-grained data on the awareness and use of HTPs. Our results showed comparable level of awareness of HTP compared to NYTS 2021 and 2022 [13–15] and slightly higher level of awareness (15–20% and 20–25% among youth and underage young adults, respectively) compared to estimates ~ 13% from studies prior to 2020 [4, 8, 9]. Nonetheless, consistent with findings from NYTS 2021 and 2022, we found limited uptake of HTPs among underage individuals (ever use < 1.5% and 5.0% among youth and underage young adults, respectively; past 30-day use < 1% in both youth and underage young adults). It is worth mentioning that a few previous studies [7–9] were conducted before the US Food and Drug Administration authorized the marketing of IQOS®, and when other HTPs were in very limited distribution (if any) in the US, while the current study was conducted after the authorization of IQOS® until it was removed from the market during quarter 4 of 2021. Taken together, results for NPs and HTPs from this study detected little uptake of these emerging tobacco products among underage individuals. Nonetheless, continued surveillance is necessary for this population.

The estimated ever and past 30-day use of e-cigarettes in the current study was comparable to those from the most recent NSDUH 2021, another household survey, which showed 12.1% and 25.1% for ever use among 12–17 year olds and 18–20 year olds, and 5.2% and 13.7% for past 30-day use [17]. Estimates for underage young adults from this study were also in line with those from the National Health Interview Survey 2021 and Population Assessment of Tobacco and Health wave 5.5 (conducted in 2020), both of which were household surveys [18, 19]. Estimates for youth from this study was lower compared to results from the estimate from school surveys, such as NYTS and Monitoring the Future (e.g., 3–6% from the current study vs. 7.6% from NYTS 2021 and 9.4% from NYTS 2022) [6, 14, 20]. We consider the following to be the main reasons for the observed differences. First, the UTUS is an address-based household survey and it has been well documented that household surveys typically produce lower estimates for underage tobacco use when compared to school surveys [21–23]. Second, the study population of the NYTS was all middle- and high-school students, of whom some were 18 years of age or older. In contrast, the estimates among youth from the UTUS were among 13–17 year olds.

The estimates observed in our study were likely influenced by the effects of the COVID-19 pandemic. A few recent studies have documented a large reduction in e-cigarette use among youth during the COVID-19 pandemic [24, 25]. Multiple factors can underlie this observed decline in tobacco use during the COVID-19 pandemic, including reduced access to retail [26], reduced access from peers, increased parental monitoring due to stay-at-home restrictions, etc. It is well documented that youth access to tobacco is largely through social sources (e.g., from friends or someone else) [27, 28] and socializing with peers was a common reason for e-cigarette use [5]. COVID-19 could serve as a barrier to this motive due to concerns about infection and reduced face-to-face contact with peers to share an e-cigarette [26] and block social access to tobacco due to reduced in-person social interaction. There is also likely increased parental monitoring during the lockdown. In addition, the federal minimum age of sale of tobacco products was raised from 18 to 21 years in December 2019, which may also restrict youth access to tobacco products, especially among 15–17 year olds [29]. The observed slight upward trend in past 30-day e-cigarette use among youth between quarter one of 2021 and quarter two of 2022 is in line with the hypothesized effects of COVID-19 as schools and the larger society gradually opened up during this period. Nonetheless, the slight increase has stopped in quarter 3 of 2022. Continued monitoring is necessary to provide timely information about youth e-cigarette use after the COVID-19 pandemic.

To the best of our knowledge, the current study is the first to report the awareness, ever use, and past 30-day use among underage 18–20 year olds in 2020–2022 Our estimates were generally in line with estimates from NHIS 2020. For example, estimated ever and current e-cigarette use among 18–20 year olds from NHIS 2020 was 28.3% and 8.3%, respectively, and our estimates ranged from 30 to 36% for ever use and 11–14% for past 30-day use. Previous studies have shown a lag between the enactment of nationwide policies and observed change in behaviors [30, 31]. Although the UTUS was launched after the federal legal age to purchase tobacco was raised to 21, estimates from the current study suggest that any effect of the increased minimum age on underage young adult tobacco use is yet to be seen.

Several limitations should be considered when interpreting results from the UTUS. First, non-household-dwelling individuals were excluded. Second, the relatively low level of response at the household level (between 6 and 9%) renders the study susceptible to potential selection biases. Nonetheless, the response levels are not distant from other surveys using similar methodology [32], and we used weights to bring sample distribution into balance with the national distribution in selected demographic characteristics, which helped mitigate the potential selection bias. Third, all assessments were based on self-reporting, which can result in under-reporting of tobacco use. Although we embedded language to encourage parents to provide privacy, it was not guaranteed. Fourth, in this initial report, we provided estimates stratified by age (i.e., youth and young adults) only in order to focus on quarterly trend over time. Future studies assessing subgroup variations by other demographic characteristics (e.g., sex, geographical region, race/ethnicity, etc.) among youth and young adults can provide further insights about demographic groups at higher risk of underage tobacco use. Last but not least, UTUS survey questions did not differentiate whether the source of nicotine was tobacco derived or synthetic. Future questions about products with synthetic nicotine will provide more information given the increasing presence of such products. Counter balancing strengths include a national coverage and a probability-based sampling scheme. The mail-push to online or phone approach does not require face-to-face contact, which facilitated data collection during the COVID-19 pandemic. Compared to household visitations, the mail-push to online approach is less intensive in terms of time and resources and enables a quarterly data collection schedule.

Taken together, findings from this study and other national surveys highlight the need for continued surveillance of tobacco use among underage individuals, including young adults 18–20 years of age. This is essential for the assessment of potential impacts of foreseeable and unforeseeable changes such as the introduction of new tobacco products, tobacco-related policies, and COVID-19. Recognizing the importance of such data, ALCS has made the UTUS publicly accessible via a data request process. Interested researchers may visit https://sciences.altria.com/library/underage-tobacco-use-survey?src=topnav for details.

## Electronic supplementary material

Below is the link to the electronic supplementary material.

Supplementary Material 1

## Data Availability

The datasets used and/or analysed during the current study are available from the corresponding author on reasonable request.

## Abbreviations

US: United States
NPs: Nicotine products
HTPs: heated tobacco products
NYTS: National Youth Tobacco Survey
ALCS: Altria Client Services
UTUS: Underage Tobacco Use Survey
IRB: Institutional Review Board
PATH: Population Assessment of Tobacco and Health

## References

[1] Office of the Surgeon General. Surgeon General’s Advisory on E-cigarette Use among Youth. In: Office SGs, editor.; 2018.

[2] United States Food and Drug Administration. Get the Latest Facts on Teen Tobacco Use 2021 [Available from: https://www.fda.gov/tobacco-products/youth-and-tobacco/get-latest-facts-teen-tobacco-use.

[3] Johnston LD, Miech RA, O’Malley PM, Bachman JG, Schulenberg JE, Patrick ME, MONITORING THE FUTURE NATIONAL SURVEY RESULTS ON DRUG USE., 1975 – 2020. Ann Arbor, MI:Institute for Social Research, The University of Michigan2021.

[4] Gentzke AS, Wang TW, Jamal A, Park-Lee E, Ren C, Cullen KA (2020). Tobacco Product Use among Middle and High School Students - United States, 2020. MMWR Morb Mortal Wkly Rep.

[5] Wang TW, Gentzke AS, Creamer MR, Cullen KA, Holder-Hayes E, Sawdey MD (2019). Tobacco Product Use and Associated factors among Middle and High School Students - United States, 2019. MMWR Surveill Summ.

[6] Park-Lee E, Ren C, Sawdey MD, Gentzke AS, Cornelius M, Jamal A (2021). Notes from the Field: E-Cigarette Use among Middle and High School Students - National Youth Tobacco Survey, United States, 2021. MMWR Morb Mortal Wkly Rep.

[7] Czoli CD, White CM, Reid JL, RJ OC, Hammond D (2020). Awareness and interest in IQOS heated tobacco products among youth in Canada, England and the USA. Tob Control.

[8] Dai H (2020). Heated tobacco product use and associated factors among U.S. youth, 2019. Drug Alcohol Depend.

[9] Azagba S, Shan L (2021). Heated Tobacco Products: awareness and ever use among U.S. adults. Am J Prev Med.

[10] Dove MS, Stewart SL, Tong EK (2021). Smoking behavior in 18–20 year-olds after tobacco 21 policy implementation in California: a difference-in-differences analysis with other states. Prev Med.

[11] Friedman AS, Wu RJ (2020). Do local Tobacco-21 laws reduce smoking among 18 to 20 Year-Olds?. Nicotine Tob Res.

[12] Friedman AS, Buckell J, Sindelar JL (2019). Tobacco-21 laws and young adult smoking: quasi-experimental evidence. Addiction.

[13] Promotion USNCfCDPaH, Atlanta GA. United States National Center for Chronic Disease Prevention and Health Promotion Office on Smoking and Health; 2023.

[14] Park-Lee E, Ren C, Cooper M, Cornelius M, Jamal A, Cullen KA (2022). Tobacco Product Use among Middle and High School Students - United States, 2022. MMWR Morb Mortal Wkly Rep.

[15] Gentzke AS, Wang TW, Cornelius M, Park-Lee E, Ren C, Sawdey MD (2022). Tobacco Product Use and Associated factors among Middle and High School Students - National Youth Tobacco Survey, United States, 2021. MMWR Surveill Summ.

[16] Marynak KL, Wang X, Borowiecki M, Kim Y, Tynan MA, Emery S (2021). Nicotine pouch unit sales in the US, 2016–2020. JAMA.

[17] United States Substance Abuse and Mental Health Services Administration. Results from the 2020 National Survey on Drug Use and Health: Detailed Tables. Center for Behavioral Health Statistics and Quality, editor. Rockville, Maryland:United States Substance Abuse and Mental Health Services Administration; 2021.

[18] Inter-university Consortium for Political and Social Research (2022). Population Assessment of Tobacco and Health (PATH) study Special Collection ICPSR Codebook for Wave 5.5: adult Questionnaire Data.

[19] United States Centers for Disease Control and Prevention. National Health Interview Survey 2021 Data, Questionnaires, and Related Documentation: United States Centers for Disease Control and Prevention National Center for Health Statistics; 2022 [Available from: https://www.cdc.gov/nchs/nhis/2021nhis.htm.

[20] Miech RA, Johnston LD, Patrick ME, O’Malley PM, Bachman JG, Schulenberg JE (2023). Monitoring the Future National Survey results on Drug Use, 1975–2022: secondary school students.

[21] Rootman I, Smart RG (1985). A comparison of alcohol, tobacco and drug use as determined from household and school surveys. Drug Alcohol Depend.

[22] Griesler PC, Kandel DB, Schaffran C, Hu MC, Davies M (2008). Adolescents’ inconsistency in self-reported smoking: a comparison of reports in School and in Household Settings. Public Opin Q.

[23] Cho B, Hirschtick JL, Usidame B, Meza R, Mistry R, Land SR (2021). Sociodemographic patterns of Exclusive, Dual, and Polytobacco Use among U.S. High School students: a comparison of three nationally representative surveys. J Adolesc Health.

[24] Wright LJ, Williams SE, Veldhuijzen van Zanten J (2021). Physical activity protects against the negative impact of Coronavirus Fear on adolescent Mental Health and Well-Being during the COVID-19 pandemic. Front Psychol.

[25] Gaiha SM, Lempert LK, Halpern-Felsher B (2020). Underage Youth and Young Adult e-Cigarette Use and Access before and during the Coronavirus Disease 2019 Pandemic. JAMA Netw Open.

[26] Kreslake JM, Simard BJ, O’Connor KM, Patel M, Vallone DM, Hair EC (2021). E-Cigarette use among youths and young adults during the COVID-19 pandemic: United States, 2020. Am J Public Health.

[27] Tanski S, Emond J, Stanton C, Kirchner T, Choi K, Yang L (2019). Youth Access to Tobacco Products in the United States: findings from Wave 1 (2013–2014) of the Population Assessment of Tobacco and Health Study. Nicotine Tob Res.

[28] Liu ST, Snyder K, Tynan MA, Wang TW (2019). Youth Access to Tobacco Products in the United States, 2016–2018. Tob Regul Sci.

[29] Bonnie RJ, Stratton K, Kwan LY. Public Health Implications of Raising the Minimum Age of Legal Access to Tobacco Products. In: Bonnie RJ, Stratton K, Kwan LY, editors. Public Health Implications of Raising the Minimum Age of Legal Access to Tobacco Products. Washington (DC)2015.

[30] Cheng HG, Anthony JC (2016). Does our legal minimum drinking age modulate risk of first heavy drinking episode soon after drinking onset? Epidemiological evidence for the United States, 2006–2014. PeerJ.

[31] Anderson DM, Rees DI, Sabia JJ, Safford S (2021). Association of Marijuana Legalization with Marijuana Use among US High School Students, 1993–2019. JAMA Netw Open.

[32] United States Substance Abuse and Mental Health Services Administration Center for Behavioral Health Statistics and Quality. 2021 National Survey on Drug Use and Health (NSDUH) Methodological Resource Book Sect. 14: Sample Experience Report In: Department of Health and Human Services, editor. Rockville, Maryland SAMSA; 2022.

