# Supplementary material for: Awareness and use of tobacco products among underage individuals: findings from the altria client services underage tobacco use survey 2020–2022
Source: BMC Public Health. 2023 Apr 7;23:662. doi: 10.1186/s12889-023-15610-1 (PMC10081293; doi:10.1186/s12889-023-15610-1)
Supplement: Supplementary file 1 — Supplementary Material 1 [file 12889_2023_15610_MOESM1_ESM.docx]

**Supplementary Material**

| **Table S1. Product Description and Survey Questions Used in the ALCS Underage Tobacco Use Survey.** | | | | |
| --- | --- | --- | --- | --- |
| Tobacco product category | Product Description | Survey questions | | |
|  |  | Awareness | Ever use | Recency of Use |
| E-Cigarettes | The next question is about electronic cigarettes or e-cigarettes, such as JUUL^®^, Vuse^®^, blu^®^, and Logic^™^. E-Cigarettes are battery-powered devices that usually contain a nicotine-based liquid that is vaporized and inhaled. You may also know them as e-cigs, vape-pens, e-hookahs, vapes or mods. | Have you ever seen or heard of e-cigarettes before this study? | Have you ever used an e-cigarette, even once or twice? | When was the last time you used an e-cigarette, even one or two times? |
| Cigarettes |  | Have you ever seen or heard of cigarettes (ones that have to be lit and burned) before this study? | Have you ever smoked a cigarette, even one or two puffs? | When was the last time you smoked a cigarette, even one or two puffs? |
| Cigars | The next question is about the use of cigars, cigarillos, or little cigars such as Swisher Sweets^®^, Black and Mild^®^, Garcia y Vega^®^, Cheyenne^™^, White Owl^®^, or Dutch Masters^®^ | Have you ever seen or heard of cigars, cigarillos, or little cigars before this study? | Have you ever smoked a cigar, cigarillo, or little cigar, even one or two puffs? | When was the last time you smoked a cigar, cigarillo, or little cigar, even one or two puffs? |
| Chewing tobacco, snuff, or dip | The following question is about the use of chewing tobacco, snuff, or dip, such as Copenhagen^®^, Grizzly^®^, Skoal^®^, or Longhorn^®^. Do not think about snus or dissolvable tobacco products when answering this question. | Have you ever seen or heard of chewing tobacco, snuff, or dip before this study? | Have you ever used chewing tobacco, snuff, or dip, even just a small amount? | When was the last time you used chewing tobacco, snuff, or dip? |
| Hookah | The next question is about smoking tobacco in a hookah, which is a type of waterpipe. Shisha (or hookah tobacco) is smoked in a hookah. | Have you ever seen or heard of smoking tobacco in a hookah or waterpipe before this study? | Have you ever smoked tobacco in a hookah or waterpipe, even one or two puffs? | When was the last time you smoked tobacco in a hookah or waterpipe, even one or two puffs? |
| Pipe tobacco | The following question is about pipes filled with tobacco (not hookah or waterpipe). | Have you ever seen or heard of pipes filled with tobacco (not hookah or waterpipe) before this study? | Have you ever used pipes filled with tobacco (not hookah or waterpipe), even just one time? | When was the last time you smoked a pipe filled with tobacco, even one or two puffs? |
| Snus | The next question is about snus, which is a type of smokeless tobacco product. It usually comes in small pouches, although some snus may be sold as loose snus. Typically, with most kinds of smokeless tobacco, you spit, but you do not need to spit when using snus. Common brands include Camel Snus^®^ or General Snus^®^. Do not think about chewing tobacco, snuff, or dip when answering this question. | Have you ever seen or heard of snus before this study? | Have you ever used Snus, such as Camel Snus or General Snus, even just one time? | When was the last time you used snus, even one or two pouches? |
| Heated tobacco products | The next question is about heated tobacco products. Some people refer to these products as “heat-not-burn” tobacco products. “Heated tobacco products” heat tobacco sticks or capsules to produce a vapor. They are different from e-cigarettes, which heat a liquid to produce a vapor. Some brands of “heated tobacco products” include IQOS^®^, glo^™^, and Eclipse^®^. | Have you ever seen or heard of “heated tobacco products” before this study? | Have you ever used a “heated tobacco product”, even just one time? | When was the last time you used a heated tobacco product, even one or two times? |
| Oral nicotine products | The next question is about “oral nicotine products”. These products might come in pouches such as on!® or Zyn™, lozenges, chewables and other oral forms. Do not think about chewing tobacco, snuff, dip, snus, or products used to quit tobacco such as nicotine gum, Nicorette® or Nicoderm® when answering this question. | Have you ever seen or heard of “oral nicotine products” before this study? | Have you ever used an oral nicotine product, even just one time? | When was the last time you used an oral nicotine product, even one or two times? |

| **Table S2. Estimated Awareness (%, 95% CI) of Tobacco Products among Underage Individuals by Age. Data from the ALCS UTUS May 2020 to August 2022.** | | | | | | | | | |
| --- | --- | --- | --- | --- | --- | --- | --- | --- | --- |
| Survey | E-Cigarettes | Cigarette | Cigar | Hookah | Pipe | Smokeless Tobacco | Snus | Heated Tobacco | Oral Nicotine |
|  | **13-17** | | | | | | | | |
| 2020 Q2 | 91.3  (88.0,93.7) | 96.7  (94.4,98.1) | 79.8  (75.9,83.2) | 60.7  (56.2,65.0) | 55.6  (51.0,60.2) | 81.0  (76.8,84.6) | 16.8  (13.8,20.3) | 15.3  (12.4,18.9) | 33.7  (29.7,38.0) |
| 2020 Q3 | 94.7  (92.0,96.5) | 96.1  (93.6,97.7) | 84.5  (80.6,87.8) | 60.8  (56.0,65.4) | 57.5  (52.5,62.3) | 82.7  (78.6,86.2) | 19.8  (16.1,24.0) | 20.7  (17.1,24.9) | 33.9  (29.4,38.7) |
| 2020 Q4 | 92.8  (91.1,94.1) | 95.8  (94.4,96.8) | 83.3  (81.0,85.4) | 60.8  (57.9,63.5) | 58.7  (55.8,61.6) | 82.3  (79.8,84.5) | 18.1  (16.1,20.4) | 19.4  (17.2,21.9) | 37.9  (35.1,40.7) |
| 2021 Q1 | 89.9  (87.1,92.2) | 94.0  (91.6,95.7) | 81.8  (78.6,84.7) | 60.2  (56.3,63.9) | 56.3  (52.4,60.2) | 80.7  (77.2,83.7) | 16.1  (13.5,19.1) | 18.8  (15.9,22.0) | 42.1  (38.2,46.1) |
| 2021 Q2 | 90.9  (88.1,93.1) | 95.6  (93.2,97.2) | 82.0  (78.5,85.1) | 59.9  (55.7,64.0) | 55.9  (51.5,60.3) | 80.6  (76.7,83.9) | 19.1  (16.0,22.7) | 2<0.1  (16.7,23.8) | 39.7  (35.7,43.9) |
| 2021 Q3 | 92.2  (89.3,94.3) | 96.4  (94.3,97.8) | 80.3  (76.7,83.5) | 55.5  (51.0,59.8) | 57.1  (52.6,61.5) | 82.2  (78.5,85.4) | 16.1  (13.2,19.6) | 16.8  (13.7,20.5) | 38.4  (34.2,42.9) |
| 2021 Q4 | 91.2  (88.3,93.4) | 95.9  (93.7,97.4) | 75.9  (71.7,79.6) | 57.9  (53.4,62.2) | 56.5 (51.9,61.0) | 73.5 (69.0,77.4) | 16.9 (13.8,20.5) | 16.8 (13.7,20.5) | 38.9 (34.7,43.2) |
| 2022 Q1 | 91.2  (88.7,93.2) | 94.7  (92.4,96.3) | 79.1  (75.6,82.3) | 55.7  (51.7,59.7) | 49.9 (45.8,53.9) | 74.5 (70.7,78.0) | 17.0 (14.2,20.2) | 17.4 (14.7,20.5) | 41.4 (37.7,45.3) |
| 2022 Q2 | 93.6  (91.3,95.3) | 96.0  (93.7,97.4) | 82.8  (79.5,85.7) | 60.3  (56.3,64.1) | 60.3 (56.3,64.3) | 81.3 (77.7,84.4) | 19.6 (16.4,23.2) | 21.6 (18.3,25.3) | 38.8 (34.8,42.9) |
| 2022 Q3 | 92.1  (89.6,94.0) | 95.5  (93.6,96.8) | 81.0  (77.7,83.9) | 55.8  (51.7,59.8) | 52.7 (48.6,56.8) | 77.7 (74.2,80.9) | 17.7 (14.9,20.8) | 13.6 (11.1,16.5) | 37.8 (33.9,41.7) |
|  | **18-20** | | | | | | | | |
| 2020 Q2 | 94.5  (90.6,96.8) | 95.4  (91.7,97.5) | 88.3  (83.1,92.1) | 85.9  (80.9,89.8) | 73.1  (67.1,78.4) | 89.1  (84.6,92.3) | 30.2  (24.9,36.1) | 22.0  (17.5,27.3) | 51.7  (45.6,57.7) |
| 2020 Q3 | 97.0  (93.4,98.7) | 96.7  (93.2,98.4) | 86.6  (80.7,90.8) | 77.7  (71.2,83.1) | 68.8  (61.7,75.2) | 91.6  (87.2,94.6) | 26.2  (20.4,33.0) | 28.5  (22.2,35.7) | 47.7  (40.6,55.0) |
| 2020 Q4 | 95.4  (93.7,96.7) | 95.5  (93.8,96.8) | 90.4  (88.1,92.3) | 81.6  (78.7,84.1) | 75.7  (72.6,78.5) | 89.3  (86.9,91.3) | 32.1  (29.0,35.4) | 29.0  (26.0,32.1) | 50.7  (47.2,54.2) |
| 2021 Q1 | 93.9  (90.8,96.0) | 95.3  (92.4,97.1) | 88.1  (84.7,90.8) | 79.5  (75.5,83.0) | 70.1  (65.8,74.1) | 89.5  (86.2,92.1) | 30.1  (26.2,34.2) | 24.6  (20.9,28.7) | 53.1  (48.7,57.4) |
| 2021 Q2 | 93.9  (90.7,96.1) | 95.1  (92.3,97.0) | 87.6  (83.8,90.5) | 78.4  (73.9,82.3) | 63.0  (58.2,67.6) | 84.8  (80.5,88.3) | 27.9  (23.6,32.5) | 23.0  (19.1,27.4) | 52.0  (46.9,57.1) |
| 2021 Q3 | 94.2  (91.0,96.4) | 92.4  (88.6,95.0) | 87.5  (83.2,90.8) | 78.1  (73.2,82.3) | 68.1  (62.9,72.9) | 87.5  (83.3,90.8) | 28.4  (24.0,33.3) | 28.2  (23.9,33.1) | 52.6  (47.3,57.8) |
| 2021 Q4 | 96.4 (94.1,97.8) | 97.1 (95.0,98.4) | 91.9 (88.7,94.2) | 84.4 (80.2,87.9) | 74.4 (69.6,78.7) | 90.8 (87.2,93.5) | 32.2 (27.5,37.2) | 27.9 (23.5,32.9) | 58.6 (53.3,63.7) |
| 2022 Q1 | 95.0 (92.5,96.7) | 94.1 (91.5,96.0) | 88.4 (85.1,91.1) | 80.9 (76.9,84.4) | 69.3 (64.8,73.4) | 83.3 (79.3,86.7) | 30.9 (26.8,35.4) | 25.9 (22.0,30.2) | 55.8 (51.0,60.4) |
| 2022 Q2 | 97.0 (95.0,98.2) | 96.7 (94.7,98.0) | 88.8 (85.2,91.6) | 78.6 (74.5,82.2) | 68.3 (63.6,72.6) | 86.1 (82.4,89.1) | 29.5 (25.4,33.8) | 29.3 (25.2,33.7) | 56.3 (51.6,61.0) |
| 2022 Q3 | 95.8 (93.4,97.3) | 96.8 (95.0,98.0) | 87.4 (83.9,90.2) | 82.9 (79.2,86.0) | 69.0 (64.6,73.1) | 88.4 (85.2,91.0) | 31.4 (27.1,36.1) | 29.1 (24.9,33.7) | 54.7 (50.0,59.4) |
| CI: confidence interval. ALCS UTUS: Altria Client Services Underage Tobacco Use Survey. | | | | | | | | | |

| **Table S3. Estimated Ever Use (%, 95% CI) of Tobacco Products among Underage Individuals by Age. Data from the ALCS UTUS May 2020 to August 2022.** | | | | | | | | | |
| --- | --- | --- | --- | --- | --- | --- | --- | --- | --- |
| Survey | E-Cigarettes | Cigarette | Cigar | Hookah | Pipe | Smokeless Tobacco | Snus | Heated Tobacco | Oral Nicotine |
|  | **13-17** | | | | | | | | |
| 2020 Q2 | 12.0  (9.5,15.1) | 5.8  (3.9,8.6) | 2.8  (1.8,4.4) | 3.6  (2.3,5.5) | 0.4  (0.1,1.1) | 2.0  (1.1,3.8) | 0.7  (0.3,1.6) | 0.6  (0.3,1.3) | 0.4  (0.1,1.5) |
| 2020 Q3 | 12.4  (9.6,16.0) | 6.4  (4.3,9.3) | 4.9  (3.2,7.6) | 1.5  (0.6,3.5) | 1.3  (0.5,2.9) | 2.3  (1.3,4.2) | 0.7  (0.3,2.0) | 1.2  (0.6,2.7) | 0.8  (0.3,2.2) |
| 2020 Q4 | 15.7  (13.6,17.9) | 8.8  (7.3,10.6) | 6.0  (4.8,7.4) | 2.2  (1.4,3.3) | 1.2  (0.7,1.9) | 1.9  (1.4,2.8) | 0.5  (0.3,1.1) | 1.2  (0.7,1.9) | 0.9  (0.5,1.5) |
| 2021 Q1 | 13.5  (11.1,16.2) | 5.8  (4.4,7.6) | 3.9  (2.7,5.6) | 2.4  (1.5,3.7) | 1.0  (0.5,2.1) | 1.5  (0.9,2.7) | 0.4  (0.1,1.1) | 1.1  (0.6,2.1) | 1.1  (0.6,2.1) |
| 2021 Q2 | 14.5  (11.8,17.6) | 7.0  (5.2,9.4) | 4.2  (2.8,6.2) | 1.9  (1.1,3.4) | 1.1  (0.5,2.2) | 2.2  (1.2,4.0) | 1.0  (0.4,2.2) | 1.5  (0.8,2.6) | 0.7  (0.3,1.8) |
| 2021 Q3 | 10.9  (8.5,13.9) | 4.9  (3.4,7.1) | 3.8  (2.4,6.0) | 2.2  (1.2,3.8) | 0.8  (0.3,2.0) | 1.0  (0.5,2.0) | 0.7  (0.3,1.8) | 1.2  (0.6,2.4) | 0.9  (0.4,2.0) |
| 2021 Q4 | 13.5 (10.8,16.8) | 7.5 (5.6,9.9) | 5.4 (3.8,7.6) | 1.9 (1.0,3.5) | 1.0 (0.4,2.5) | 2.3 (1.3,3.9) | 0.8 (0.3,2.2) | 0.8 (0.3,2.4) | 1.4 (0.7,3.1) |
| 2022 Q1 | 14.9 (12.3,18.0) | 7.4 (5.5,9.9) | 5.8 (4.2,8.1) | 3.3 (2.2,4.9) | 0.6 (0.2,1.6) | 1.7 (1.0,3.0) | 1.0 (0.5,2.0) | 1.1 (0.6,2.2) | 1.6 (0.9,2.9) |
| 2022 Q2 | 14.9 (12.2,18.1) | 6.8 (5.1,9.0) | 4.0 (2.7,6.0) | 4.2 (2.8,6.5) | 1.4 (0.7,2.8) | 2.8 (1.8,4.3) | 0.9 (0.4,2.0) | 1.9 (1.1,3.4) | 1.3 (0.6,2.5) |
| 2022 Q3 | 12.8 (10.4,15.6) | 8.4 (6.4,11.0) | 5.1 (3.7,7.1) | 2.3 (1.4,3.9) | 0.7 (0.3,1.6) | 2.0 (1.2,3.4) | 1.0 (0.5,2.2) | 1.8 (1.0,3.0) | 1.8 (1.0,3.4) |
|  | **18-20** | | | | | | | | |
| 2020 Q2 | 31.5  (25.9,37.7) | 20.7  (15.9,26.6) | 20.7  (15.8,26.6) | 16.0  (11.5,21.8) | 3.1  (1.6,5.8) | 7.8  (5.1,11.8) | 2.6  (1.3,5.2) | 2.0  (1.0,4.0) | 5.0  (3.1,8.1) |
| 2020 Q3 | 30.2  (23.9,37.2) | 19.8  (15.0,25.8) | 16.9  (12.3,22.7) | 9.6  (6.3,14.3) | 2.3  (1.0,4.8) | 2.4  (1.0,5.4) | 1.6  (0.6,4.8) | 3.6  (1.7,7.5) | 2.9  (1.2,6.8) |
| 2020 Q4 | 36.0  (32.8,39.4) | 25.6  (22.7,28.6) | 18.8  (16.3,21.6) | 14.6  (12.3,17.3) | 3.7  (2.6,5.1) | 6.0  (4.7,7.8) | 2.1  (1.4,3.2) | 4.2  (3.0,5.7) | 3.5  (2.6,4.9) |
| 2021 Q1 | 31.8  (27.9,36.0) | 20.2  (16.9,23.9) | 16.5  (13.5,20.1) | 9.6  (7.5,12.3) | 4.2  (2.7,6.5) | 5.3  (3.8,7.5) | 1.6  (0.9,2.9) | 2.9  (1.7,4.8) | 3.5  (2.1,5.6) |
| 2021 Q2 | 30.9  (26.5,35.7) | 19.3  (15.6,23.7) | 15.2  (11.8,19.4) | 10.6  (7.9,14.0) | 3.5  (2.0,6.1) | 4.4  (2.6,7.4) | 2.0  (0.8,5.1) | 3.1  (1.7,5.5) | 2.1  (1.2,3.8) |
| 2021 Q3 | 28.9  (24.4,33.8) | 18.4  (14.7,22.9) | 15.9  (12.6,19.9) | 9.9  (7.3,13.3) | 3.4  (2.0,5.6) | 6.0  (4.0,9.1) | 2.7  (1.5,4.8) | 3.4  (2.1,5.7) | 5.2  (3.2,8.4) |
| 2021 Q4 | 32.5 (27.9,37.5) | 20.8 (17.0,25.2) | 17.7 (14.1,22.0) | 11.9 (8.8,15.9) | 3.0  (1.7,5.2) | 5.5 (3.7,8.1) | 1.4 (0.7,3.0) | 2.4 (1.3,4.2) | 3.3 (1.8,5.9) |
| 2022 Q1 | 33.6 (29.3,38.1) | 21.4 (17.9,25.4) | 18.1 (14.8,22.0) | 12.8 (9.8,16.4) | 3.6  (2.3,5.6) | 3.7 (2.4,5.6) | 1.1 (0.5,2.3) | 4.3 (2.8,6.6) | 5.8 (4.1,8.1) |
| 2022 Q2 | 35.3 (30.8,40.0) | 23.2 (19.3,27.6) | 19.1 (15.5,23.2) | 11.2 (8.5,14.5) | 5.6  (3.7,8.3) | 4.9 (3.3,7.3) | 1.9 (1.1,3.5) | 4.5 (2.9,6.9) | 4.3 (2.8,6.4) |
| 2022 Q3 | 35.6 (31.2,40.2) | 23.7 (19.8,28.1) | 18.3 (14.9,22.2) | 12.4 (9.5,16.0) | 4.6  (3.0,7.1) | 4.2 (2.8,6.3) | 3.4 (1.9,5.9) | 4.5 (2.8,7.1) | 6.4 (4.3,9.4) |
| CI: confidence interval. ALCS UTUS: Altria Client Services Underage Tobacco Use Survey. | | | | | | | | | |

| **Table S4. Estimated Past 30-day Use (%, 95% CI) of Tobacco Products among Underage Individuals by age. Data from the ALCS UTUS May 2020 to August 2022.** | | | | | | | | | |
| --- | --- | --- | --- | --- | --- | --- | --- | --- | --- |
| Survey | E-Cigarettes | Cigarette | Cigar | Hookah | Pipe | Smokeless Tobacco | Snus | Heated Tobacco | Oral Nicotine |
|  | **13-17** | | | | | | | | |
| 2020 Q2 | 3.7 (2.5,5.6) | 1.1 (0.5,2.6) | 0.6 (0.2,1.8) | 0.6 (0.2,1.6) | 0.1 (<0.1,0.9) | 0.4 (0.1,1.1) | 0.2 (<0.1,1.4) | <0.1 (<0.1,<0.1) | 0.2 (<0.1,1.4) |
| 2020 Q3 | 2.7 (1.6,4.7) | 0.5 (0.1,3.0) | 0.6 (0.2,1.8) | 0.2 (<0.1,1.6) | 0.2 (<0.1,1.7) | 0.2 (<0.1,1.1) | 0 (.,.) | 0 (.,.) | 0.4 (0.1,1.5) |
| 2020 Q4 | 4.1 (3.1,5.3) | 1.2 (0.7,1.9) | 1.5 (0.9,2.2) | 0.7 (0.3,1.5) | 0.2 (0.1,0.4) | 0.3 (0.1,0.8) | 0.2 (<0.1,0.7) | 0.2 (0.1,0.6) | 0.2 (0.1,0.6) |
| 2021 Q1 | 3.0 (2.0,4.5) | 1.3 (0.7,2.4) | 1.0 (0.5,2.2) | 0.4 (0.1,0.9) | 0 (.,.) | 0.1 (<0.1,1.0) | 0.1 (<0.1,1.0) | 0.4 (0.2,1.1) | 0.2 (0.1,0.8) |
| 2021 Q2 | 3.3 (2.1,5.2) | 1.0 (0.5,2.1) | 0.9 (0.3,2.2) | 0.1 (<0.1,0.5) | 0 (.,.) | 0.2 (<0.1,1.3) | 0.2 (<0.1,1.4) | 0.2 (<0.1,1.2) | 0.1 (<0.1,0.9) |
| 2021 Q3 | 3.4 (2.2,5.3) | 0.6 (0.2,1.9) | 1.9 (0.9,3.8) | 0.2 (0.1,0.8) | 0.1 (<0.1,0.4) | <0.1 (.,.) | 0.1 (<0.1,0.4) | 0.2 (<0.1,0.8) | 0.2 (<0.1,0.7) |
| 2021 Q4 | 3.8 (2.6,5.7) | 0.7 (0.3,1.5) | 1.3 (0.6,2.7) | 0.6 (0.2,1.9) | 0 (.,.) | 0.4 (0.1,1.1) | 0.3 (0.0,2.0) | 0 (.,.) | <0.1 (0.0,0.2) |
| 2022 Q1 | 4.5 (3.2,6.4) | 1.3 (0.7,2.7) | 1.4 (0.7,2.8) | 0.9 (0.4,1.9) | 0.4 (0.1,1.4) | 0.4 (0.1,1.2) | 0.3 (0.1,1.1) | 0.4 (0.1,1.2) | 0.3 (0.1,1.0) |
| 2022 Q2 | 5.7 (3.9,8.3) | 1.1 (0.5,2.3) | 1.6 (0.8,3.0) | 0.5 (0.2,1.4) | 0.4 (0.1,2.0) | 0.6 (0.2,1.5) | 0 (.,.) | 0.3 (0.1,1.4) | 0.1 (<0.1,0.5) |
| 2022 Q3 | 5.0 (3.6,6.8) | 1.1 (0.6,2.1) | 1.4 (0.7,2.7) | 0.4 (0.1,1.1) | 0.1 (<0.1,0.7) | 0.6 (0.2,1.7) | 0.1 (<0.1,0.9) | 0.1 (<0.1,0.7) | 0.4 (0.1,1.3) |
|  | **18-20** | | | | | | | | |
| 2020 Q2 | 11.0 (7.7,15.5) | 3.9 (2.2,6.8) | 4.6 (2.3,8.9) | 2.2 (0.9,5.3) | 1.0 (0.3,2.9) | 1.3 (0.6,3.0) | 0.9 (0.3,2.7) | 0.1 (<0.1,0.9) | 1.4 (0.6,3.7) |
| 2020 Q3 | 11.8 (7.9,17.2) | 4.5 (2.4,8.3) | 3.7 (1.9,7.2) | 3.3 (1.5,7.4) | 0.5 (0.1,2.0) | 1.1 (0.3,4.2) | <0.1 (<0.1,0.1) | <0.1 (<0.1,0.1) | 0.7 (0.2,2.9) |
| 2020 Q4 | 14.2 (11.9,16.8) | 5.6 (4.2,7.3) | 5.3 (3.9,7.0) | 3.6 (2.5,5.2) | 1.3 (0.7,2.3) | 0.8 (0.4,1.6) | 0.4 (0.2,1.1) | 1.0 (0.5,1.9) | 0.8 (0.4,1.5) |
| 2021 Q1 | 11.6 (9.0,14.8) | 4.0 (2.6,6.2) | 4.1 (2.7,6.1) | 0.4 (0.1,0.9) | 0.4 (0.1,1.4) | 0.8 (0.3,2.2) | 0 (.,.) | 0.5 (0.2,1.3) | 0.6 (0.2,1.8) |
| 2021 Q2 | 11.2 (8.3,15.0) | 2.4 (1.2,4.4) | 2.0 (0.9,4.2) | 1.4 (0.6,3.1) | 1.0 (0.4,2.9) | 0.3 (0.1,1.3) | 0 (.,.) | 0 (.,.) | 0.7 (0.2,2.0) |
| 2021 Q3 | 11.5 (8.5,15.4) | 3.8 (2.1,6.6) | 4.2 (2.6,6.8) | 1.9 (0.9,4.0) | 0.7 (0.2,2.5) | 1.2 (0.4,3.4) | 0.2 (0.1,1.1) | 0.8 (0.3,2.0) | 1.7 (0.6,4.3) |
| 2021 Q4 | 10.4 (7.8,13.8) | 4.0 (2.4,6.6) | 4.4 (2.6,7.2) | 2.2 (1.1,4.6) | 0.7 (0.2,2.3) | 1.3 (0.6,2.7) | 0.0 (0.0,0.2) | 0.7 (0.3,2.0) | 0.9 (0.2,4.3) |
| 2022 Q1 | 13.2 (10.4,16.7) | 5.6 (3.8,8.2) | 5.6 (3.7,8.4) | 1.6 (0.7,3.7) | 0.7 (0.3,1.9) | 0.4 (0.1,1.1) | 0.1 (0.0,0.6) | 0.6 (0.2,1.6) | 1.0 (0.4,2.6) |
| 2022 Q2 | 12.0 (9.3,15.4) | 4.6 (2.7,7.7) | 6.3 (4.2,9.4) | 2.4 (1.3,4.3) | 0.6 (0.2,1.5) | 0.4 (0.1,1.9) | 0.5 (0.1,1.9) | 1.5 (0.7,3.0) | 1.2 (0.6,2.6) |
| 2022 Q3 | 12.5 (9.7,16.0) | 4.8 (3.0,7.6) | 3.6 (2.2,5.9) | 2.1 (1.1,4.0) | 1.3 (0.5,3.0) | 0 (.,.) | 0.6 (0.2,1.9) | 0.7 (0.2,1.9) | 1.3 (0.6,2.7) |
| CI: confidence interval. ALCS UTUS: Altria Client Services Underage Tobacco Use Survey. | | | | | | | | | |
